# Revitalization of integrated disease surveillance and response in Sierra Leone post Ebola virus disease outbreak

**DOI:** 10.1186/s12889-019-6636-1

**Published:** 2019-04-02

**Authors:** Charles Njuguna, Amara Jambai, Alexander Chimbaru, Anders Nordstrom, Roland Conteh, Anderson Latt, Shikanga O-tipo, Robert Musoke, Jane Githuku, Zablon Yoti, Ali Yahaya, Ambrose Talisuna, Soatiana Rajatonirina, Ibrahima Socé Fall

**Affiliations:** 1 World Health Organization, Freetown, Sierra Leone; 2 Ministry of Health and Sanitation, Freetown, Sierra Leone; 3 World Health Organization Regional Office for Africa, Brazzaville, Congo; 4 Ministry for Foreign Affairs, Stockholm, Sweden

**Keywords:** Surveillance, Public health, Health information systems, Disease outbreak, Ebola virus disease

## Abstract

**Background:**

The Ministry of Health and Sanitation (MOHS) in Sierra Leone partially rolled out the implementation of Integrated Disease Surveillance and Response (IDSR) in 2003. After the Ebola virus disease outbreak in 2014–2015, there was need to strengthen IDSR to ensure prompt detection and response to epidemic-prone diseases. We describe the processes, successes and challenges of revitalizing public health surveillance in a country recovering from a protracted Ebola virus disease outbreak.

**Methods:**

The revitalization process began with adaptation of the revised IDSR guidelines and development of customized guidelines to suit the health care systems in Sierra Leone. Public health experts defined data flow, system operations, case definitions, frequency and channels of reporting and dissemination. Next, phased training of IDSR focal persons in each health facility and the distribution of data collection and reporting tools was done. Monitoring activities included periodic supportive supervision and data quality assessments. Rapid response teams were formed to investigate and respond to disease outbreak alerts in all districts.

**Results:**

Submission of reports through the IDSR system began in mid-2015 and by the 35th epidemiologic week, all district health teams were submitting reports. The key performance indicators measuring the functionality of the IDSR system in 2016 and 2017 were achieved (WHO Africa Region target ≥80%); the annual average proportion of timely weekly health facility reports submitted to the next level was 93% in 2016 and 97% in 2017; the proportion of suspected outbreaks and public health events detected through the IDSR system was 96% (*n* = 87) in 2016 and 100% (*n* = 85) in 2017.

**Conclusion:**

With proper planning, phased implementation and adequate investment of resources, it is possible to establish a functional IDSR system in a country recovering from a public health crisis. A functional IDSR system requires well trained workforce, provision of the necessary tools and guidelines, information, communication and technology infrastructure to support data transmission, provision of timely feedback as well as logistical support.

**Electronic supplementary material:**

The online version of this article (10.1186/s12889-019-6636-1) contains supplementary material, which is available to authorized users.

## Background

Public health surveillance generates information needed to assess health status of populations, track events of public health importance such as outbreaks, identify priorities and evaluate the effectiveness of programs [1]. Integration of disease surveillance systems is recommended to increase cost effectiveness, efficiency and effectiveness of surveillance system [2, 3]. In 1998, the WHO-Africa region countries adopted the IDSR for the timely detection and response to epidemic prone diseases [4]. The strategy proposed efficient use of resources to develop an integrated surveillance and response system for major communicable diseases as prioritized by individual countries. The IDSR strategy focused on surveillance at the district level but also defined core and support functions for other levels of the public health system. Technical guidelines for the implementation of IDSR strategy were developed in 2001 and adopted by various African countries including Sierra Leone in 2008 [5].

Prompted by the severe acute respiratory syndrome pandemic at the beginning of the twenty-first century, the re-emergence of infectious diseases and threat of the misuse of infectious agents such as smallpox virus as biological weapons, WHO member states adopted the revised International Health Regulations in 2005 which came into force in June 2007 [6]. These new regulations widened the scope of reportable events by defining public health events of international concern, core surveillance and response capacities, that countries would need to focus on in order to increase their ability to detect, respond and contain public health emergencies. Full implementation of international health regulations 2005 would ensure containment of public health threats with minimal interference [7] with international trade and travel.

In order to comply with the new regulations, WHO African member states with technical support from United States Centers for Disease Control and Prevention (CDC) revised guidelines for implementation of IDSR in 2010 [7]. Systematic reviews of the implementation of IDSR strategy shows that most African countries adopted the IDSR strategy albeit partially. However, most countries have poor performance in IDSR core functions which is closely linked to suboptimal supervision, training, resources and coordination [8].

The Ministry of Health and Sanitation (MOHS) in Sierra Leone partially rolled out the implementation of IDSR in 2003. Before the Ebola virus disease outbreak that occurred in 2014, the country had not yet adopted or implemented the revised IDSR guidelines (2010). Thus, public health surveillance was weak in Sierra Leone and may have contributed to the delayed case detection of the first Ebola cases [9]. The situation was aggravated by the infection of 328 health care workers with Ebola virus disease and the death of at least 152 [10]. Many volunteer health care workers, who constitute a considerable proportion of health workforce in Sierra Leone, resigned during the outbreak further worsening the shortage of health care workers. This led to the closure of health facilities thus interfering with collection and transmission of surveillance data. A rapid assessment conducted in 2015 showed that less than half of the health facilities were submitting weekly reports of priority diseases and often, the reports were submitted late. Thus, the surveillance system was in need of revitalization to ensure rapid detection of outbreaks and tracking of the progress of health events such as the Ebola virus disease outbreak. This paper describes the processes, successes and challenges of revitalizing public health surveillance in a country recovering from a protracted Ebola virus disease outbreak. It aims to provide insight on strategies and requirements for successful implementation of IDSR. By demonstrating the usefulness of IDSR as an early warning system for epidemic prone diseases such as Ebola Virus disease, this work hopes to trigger more investment in public health surveillance. It can provide a reference for countries/regions seeking to strengthen weak disease surveillance and response systems.

## Methods

### Adaptation of WHO African region -IDSR (2010) technical guidelines

The first step towards revitalization of IDSR in Sierra Leone involved the adaptation of the WHO AFRO IDSR guidelines of 2010 to suit the organizational structure and needs of the Sierra Leone healthcare system [11]. The Ministry of Health and Sanitation organized a five day workshop that brought together 50 experts from MOHS, WHO, CDC and other stakeholders to develop the first IDSR technical guidelines for Sierra Leone. Working in small groups, the experts discussed each chapter customizing it to suit the local context of Sierra Leone. The team identified priority diseases, conditions and public health events for inclusion in the surveillance system, and also aligned the guidelines to the international health regulations requirements.

There are eight core functions of IDSR that are performed by various levels in the health system. The first is case and event identification using standard case definitions. This is followed by case reporting to the next level, either immediately or at predefined intervals. These two functions usually occur in health facilities. Next data is collated, analyzed and interpreted at the health facility, district and national levels. Suspected cases, events and outbreaks are investigated and where possible confirmed in the laboratory. Investigations also identify the source and modes of transmission to inform control and prevention measures. Another core function at levels is preparedness to respond to future outbreaks. A high level of preparedness facilitates a well-coordinated and effective response, in case of an outbreak. District health teams and national surveillance offices provide regular feedback to data providers and other stakeholders on outcome of investigations and response activities. Periodic assessment of the effectiveness of the IDSR, including timeliness, data quality and overall performance is done with the aim of identifying and correcting gaps.

For each IDSR core function, detailed procedures, tools and guides were developed (Table 1). During discussions in a subsequent meeting, the same group of experts validated the updated guidelines. Finally, the guidelines were peer reviewed by a WHO IDSR expert and adopted for use in Sierra Leone. The guidelines provided a blueprint for implementation of the IDSR strategy in Sierra Leone by outlining the roles of the community, peripheral health units, district health management teams and the national surveillance program in IDSR.

**Table 1:** Summary of tools provided in adopted IDSR guidelines, Sierra Leone, 2015

IDSR Function	Tool/Template
Identify Cases of Priority Diseases and Events	1. Sierra Leone standard case definitions for reporting suspected priority diseases conditions and events from the health facility to the district
	2. Key signs and symptoms for case definitions for use at community level
	3. Template for identifying district reporting sites
	4. Laboratory functions by health system level
	5. List of national laboratories for confirming priority diseases and conditions
Report Priority Diseases, Conditions and Events	1. IDSR case-based reporting form (immediate reporting)
	2. IDSR health facility line listing form
	3. IDSR case-based laboratory reporting form
	4. IHR (2005) decision instrument
	5. IDSR weekly summary reporting form
	6. Report completeness (to be filled only by District & National Level)
	7. IDSR weekly/monthly summary reporting form
	8. Sierra Leone IDSR Reports and Data Sharing Log book
Analyse Data	1. Data Analysis Plan Template
	2. Guide on how to generate a line graph manually
Investigate Suspected Outbreaks and other public health events	1. District log of suspected outbreaks and rumours
	2. Checklist of laboratory supplies for use in an outbreak investigation
	3. Recommended list of personal protective equipment
	4. Guide for records review
	5. Contacts recording sheet
	6. Contact tracing form (follow-up)
Prepare to Respond to Outbreaks	1. Essential stock items for responding to outbreaks
	2. Stock situation report
	3. IDSR stock item transaction and balance sheet
Respond to Outbreaks	1. Case management guidelines
	2. Infection prevention and control guidelines
	3. Planning guide for supplemental vaccination activities and recommended immunization practices guides
	4. Guide for communication during an outbreak
Communicate Information	1. Sample district outbreak report 2. Sample public health bulletin
Monitor, evaluate surveillance and response	1. List of core indicators for the health facility level
	2. Chart for monitoring performance of IDSR indicators at health facility level
	3. List of core indicators for the district level

### Defining flow of surveillance data in IDSR system, Sierra Leone

**Fig. 1:**
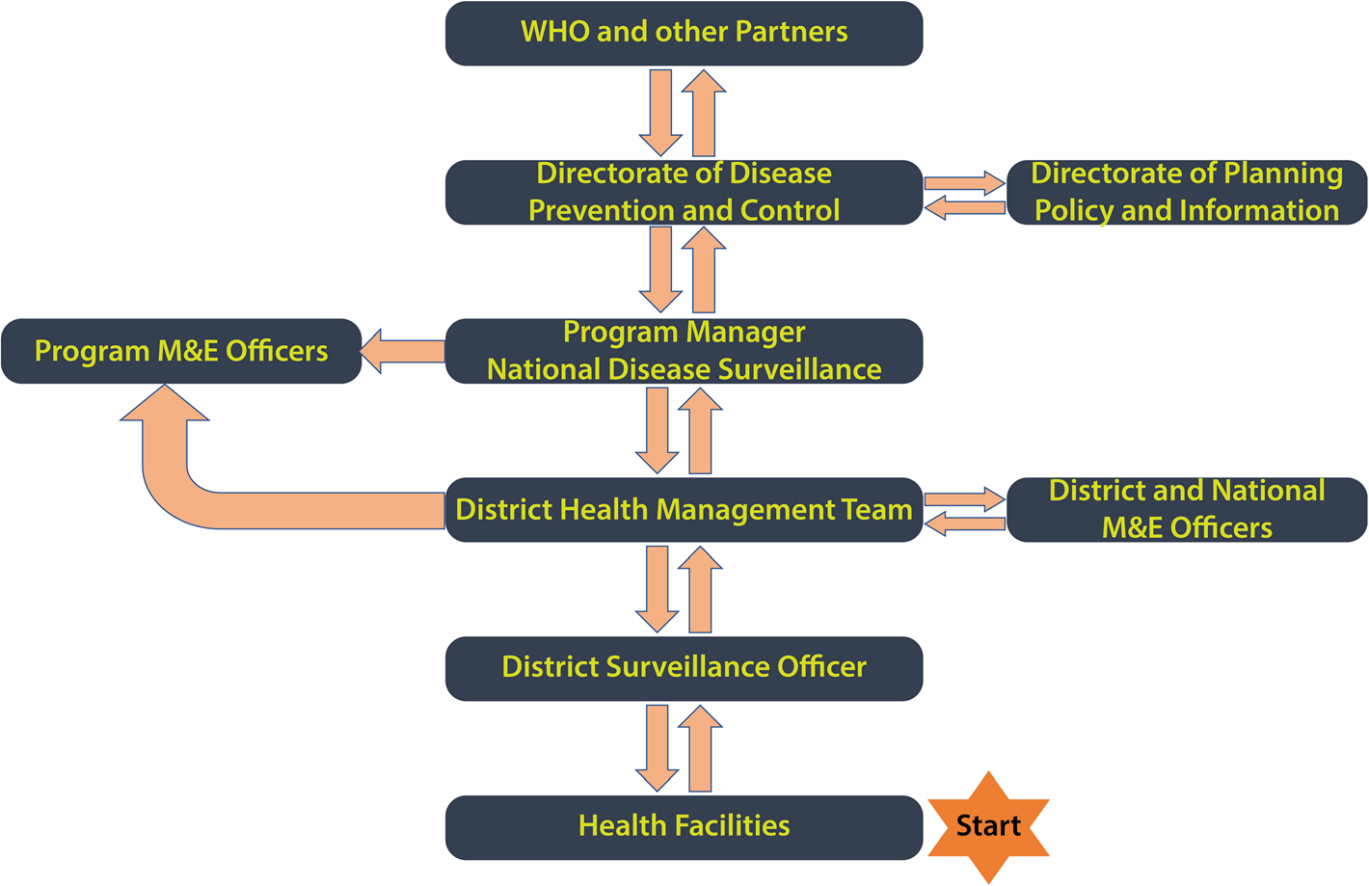
Flow chart showing data flow in IDSR system, Sierra Leone

Most public health surveillance systems combine event based surveillance at the community level and indicator based surveillance in the health facilities. In the indicator based surveillance systems, clinical and socio-demographic information collected from patients seeking treatment in health facilities is collated and transmitted to regional health departments and finally to the national international health regulations focal point who then communicates with the WHO international health regulations focal point according to international health regulations (2005) regulations [11]. Data flow in the revised IDSR guidelines for Sierra Leone was adapted to fit this outline (Fig. 1). In the proposed indicator based surveillance system, healthcare workers in peripheral health unit record patient information into registers routinely. Priority diseases would be reported every week, with the epidemiologic week starting on Monday and ending on Sunday of every week. At the end of each epidemiologic week, a surveillance focal person summarized data on priority diseases into weekly reports and forwarded them to a designated surveillance focal person in the district health office. For diseases that required immediate notification to the next level, case based forms were used for reporting. At the district health office, data from multiple peripheral health units was cleaned, checked for inconsistencies, aggregated and entered into the district health information software database which was accessed real time at the national level.

If there was a condition that warranted notification, the international health regulations focal point in the Ministry of Health and Sanitation was responsible for communicating with the WHO international health regulations focal point. Data analysis and use was encouraged at all levels, and varied from simple descriptive analysis to in-depth analysis at the national level. For example, it was expected that all health facilities would maintain a list of common diseases in their catchment populations, districts generate trends from the merged data from reporting peripheral health units and at the national level, in-depth analysis of weekly data was done with feedback given through the weekly epidemiological bulletin. Surveillance data was discussed on weekly basis in the Emergency Preparedness, Resilience and Response Group meetings at National level and in monthly meetings for all the health facility in-charges organized by the District Medical Officer in all districts.

### IDSR roll- out trainings

**Fig. 2:**
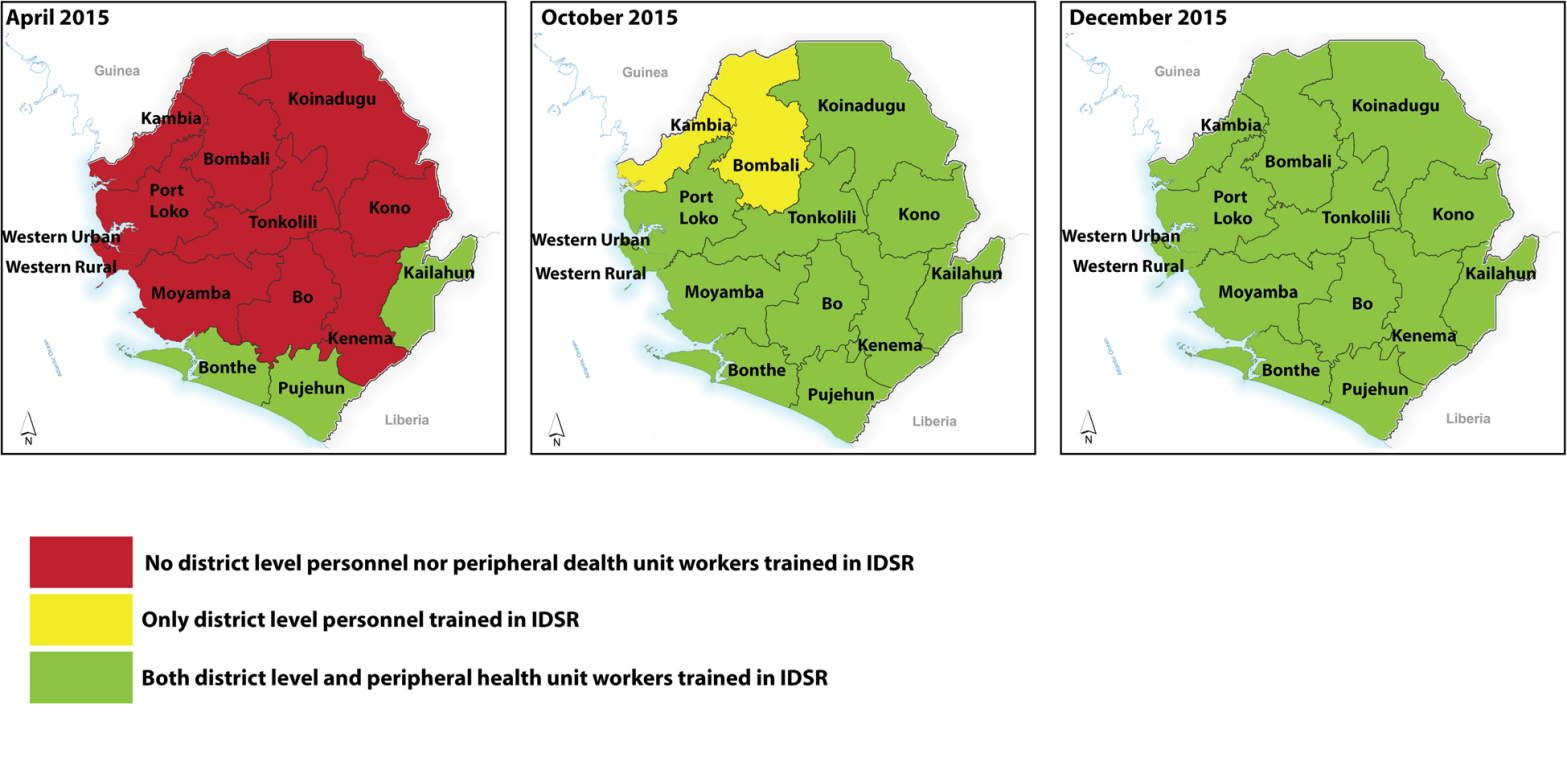
Phased roll out of IDSR trainings in Sierra Leone, 2015

A training curriculum was developed based on the adopted IDSR technical guidelines for Sierra Leone (2015). Next, the IDSR trainings were conducted in phases, beginning with training of district level trainers, then health care workers from the peripheral health units. In order to ensure full participation in all districts, healthcare workers from districts with interrupted transmission of Ebola virus disease for more than 60 days were trained first (Fig. 2). This also allowed the trainers to use the health care workers experiences from the Ebola outbreak to enhance training. Four advanced level trainings, lasting five days, were held for 144 trainers Trainer of Trainees. For each district, the trainer of trainees comprised of the district medical officer, Medical Superintendent, two district surveillance officers) and the hospital nursing officer in-charge were trained as trainer of trainees.

The IDSR trainings were organized into modules through which, participants were introduced to the concept of disease surveillance. The modules included: 1) early detection of priority diseases and other health events; 2) reporting of public health events; 3) analysis and interpretation of data generated through the surveillance system; 4) early detection of unusual changes in disease occurrence; 5) investigation and control of outbreaks, 6) response to outbreaks and other public health events; 7) communication and 8) monitoring and evaluation to improve surveillance and response. Training materials included; IDSR facilitators guide, participant’s manual, Sierra Leone IDSR Technical guidelines (2015) as well as the IDSR reporting tools. Participants were taught how to identify cases of priority diseases using standard case definitions, reporting requirements for priority diseases, data quality and data analysis and use. Presentations were used to introduce the participants to each module, then they would work through case studies in group discussions which included exercises on using data collection and reporting tools.

After each trainer of trainees training, the district trainers would be supported both financially and technically to conduct trainings for health care workers working in peripheral health units in their districts. The training for healthcare workers were less intense than the trainer of trainee trainings and focused on three modules, namely detection and reporting of priority diseases and public health events and data analysis. Health care workers in peripheral health units performed less IDSR core functions compared to those at the district level. Hence, there training was less intense.

### Supporting IDSR contributes to a strengthened healthcare system

To support IDSR, the MOHS distributed IDSR patient registers, data reporting tools, IDSR guidelines and standard case definitions posters to all district health offices and peripheral health units All district health offices were supplied with computers connected to the internet through modems.

A closed user group was created whereby IDSR focal persons, health facility in-charges and members of the District Health Management teams were connected to each other using toll free mobile phone lines. In this system, two mobile network service providers were identified and allocated districts in which to provide the service on the basis of their network coverage. One mobile line was connected in each peripheral health unit and 10 mobile phone lines for each district health management team. Similar arrangements were made for officials working at the national level in the directorate of Disease Prevention and Control and the Central Public Health Reference Laboratory. Members of the closed user group could make unlimited calls to any member of the group free of charge. This enhanced notification of disease outbreaks and also improved referrals. Each district health office had a dedicated vehicle for surveillance while a motorcycle was available in each chiefdom.

To increase human resource capacity, MOHS with support from partners also hired data clerks to assist the district surveillance officer in data management in each district health office.

### Shifting to electronic transmission of data

The IDSR data reporting system was paper based from the health facility up to the national level. Through MOHS partnership with WHO, CDC and e-Health Africa, an electronic platform was designed and rolled out in all districts in phase one. The migration was achieved through a collaborative, phased approach, led by a multidisciplinary steering committee. First, system requirements and the roles of different actors were defined. The system was anchored onto the existing District Health Information Software platform. Migration began with IDSR data at the district health office in three pilot districts, namely Port Loko, Western Area Rural and Western Area Urban and later on was implemented in all district health offices. The criteria for selecting the three districts to pilot the e-IDSR were internet connectivity and accessibility. The functioning of e-IDSR was dependent on strong internet connectivity thus the three districts with the best connectivity were selected. Additionally, there was a need for intense supervision during the initial stages. Thus, the most ideal pilot sites had to be easily accessible to allow for frequent monitoring visits. In the second phase, electronic data transmission in health facilities was tested in Port Loko district.

### Monitoring implementation of IDR through regular support supervision and data quality assessments

Starting February 2016, teams from MOHS, WHO and other partners conducted supervision visits in randomly selected peripheral health units in all districts. To improve management of data collected during the visits, a structured electronic checklist, uploaded onto the Open Data Kit (ODK) platform was used for data collection. The visits sought to assess the adequacy of staff, infrastructure, and supplies to support implementation of IDSR as well as the performance of selected IDSR indicators as per existing guidelines. The reliability of information generated through a surveillance system depends on the quality of the data collected and transmitted through the system. Cognizant of the need to ensure high quality of IDSR data, MOHS with technical support from WHO and other partners conducted periodic data quality assessments in randomly selected PHUs in the districts. A structured checklist developed using the open data kit platform and loaded onto hand held devices was used for data collection. We calculated verification factors (VF) from recounted values of malaria positive cases recorded in health facility registers and compared them to values abstracted from health facility weekly reports and the DHIS 2 database. A VF < 100 was over reporting while a VF > 100 was underreporting.

### Improving emergency preparedness and response capacity

Rapid response teams, comprising of health officials from the district health office, and WHO officials were formed in each district, to investigate and respond to suspected outbreaks. To improve emergency preparedness and response a national emergency preparedness plan was developed. Implementation of this plan resulted in the formation of multidisciplinary emergency preparedness and response teams in each district.

## Results

### Capacity building

From March 2015 to March 2016, 2300 health care workers from 14 districts were trained in IDSR, most of whom were health workers in-charges of peripheral health units. This ensured that all health facilities had at least one person trained on IDSR. Weekly reporting of priority diseases commenced immediately after each training with more health facilities submitting reports over time. Additionally, a separate training was conducted for 418 clinicians drawn from hospitals (pubic, private and faith based) all over the country. This training introduced the clinicians to the concept of disease surveillance and the role they played in generating quality surveillance data.

### Shift from the paper based reporting to electronic surveillance system (e-IDSR)

Submission of paper based reports through the IDSR system began in Mid-2015 as more surveillance focal persons were trained. By the 35th epidemiologic week, the MOHs was receiving weekly reports from all districts in the country.

Starting July 2016, IDSR data was entered into an electronic database directly from each district. Full integration of e-IDSR into the district health information software platform was completed on the fifth epidemiologic week in 2017 and this system has since been used to transmit and store IDSR data effectively. In the e-IDSR system, health facilities submit weekly reports through phone calls, mobile phone text messages, delivery of hard copies or by email to the district health office by 12 noon every Monday. After receiving the weekly reports (hard copies, text messages, phone call data), the district surveillance officer enters the data into the e-IDSR platform through the computer desktop application. The district surveillance officer reviews the data entered into the district health information software database for completeness, timeliness and validity. Real time data is accessed by the national level after the validation by the district health office. The proportion of districts with complete health facility weekly reports increased from 78% before introduction of e-IDSR to 96% after migration to e-IDSR. In 2017, Sierra Leone embarked on roll out of e-IDSR to the health facility level (phase 2) using tablets.

### Use of weekly epidemiological bulletins as a feedback mechanism

In order to provide structured feedback and disseminate the data from the surveillance system, the MOHS with technical expertise from WHO developed a weekly epidemiological bulletin (Additional file 1). The bulletin provided a summary of data on the key notifiable diseases from the weekly reports submitted by the districts. It also included analysis on the proportion of health facilities submitting weekly reports to the district and as well as timeliness of submitted reports. Completeness of reporting was measured by the proportion of health facilities that submitted weekly reports compared to the total number of health facilities expected to submit reports in a particular district. Reporting rates below 80% were considered sub-optimal as per WHO standards. This provided a measure of representativeness of the surveillance data and helped identify silent health facilities or districts for immediate follow up.

The epidemiological bulletin was circulated to all relevant district and national MOHS staff, partners including WHO. Besides serving as an effective feedback mechanism, the method of using bulletins also improved completeness and timeliness of reporting as districts with low reporting rates and late submissions were followed up to resolve challenges that contributed to poor performance. Overall, the proportion of peripheral health unit submitting weekly reports to the districts increased from 68.2% in 2015, 92.4% in 2016 and 97.3% in 2017. With time, the method became so acceptable to the district teams that several of them adopted the bulletin to provide feedback to the peripheral health units on trends of common diseases, data quality and updates on outbreaks occurring within the districts.

### Outbreak detection, notification and response to suspected outbreaks and other important public health events

The proportion of outbreaks detected through the IDSR system increased from 96% in 2016 to 100% in 2017. Laboratory confirmation of outbreaks also improved with 72% of laboratory results received within a week in 2017 compared to 17% in 2016. Timeliness in notification of and response to outbreaks declined from 92% in 2016 to 81% in 2017 (Table 2). The likely reasons for the decline were reduced WHO logistical support and staffing levels providing support to the district health management teams in 2017. Starting 2015, through 2017, WHO scaled down its presence in all districts as part of the post Ebola recovery plan.

**Table 2:** Detection, notification, and response performance indicators, Sierra Leone 2016–2017

Indicator	2016	2017
Number of suspected outbreaks & public health (PH) events reported from all sources	87	85
Proportion of suspected outbreaks or public health events detected through IDSR system	96%	100%
Proportion of suspected outbreaks or public health events notified within 24 h	92%	81%
Proportion of suspected outbreaks or public health events with rapid response within 48 h	90%	87%
Proportion of investigated outbreaks or public health events with lab results within 7 days	17% (*n* = 58)	72% (*n* = 46)

### Measles and rubella outbreak 2016–2017

**Fig. 3:**
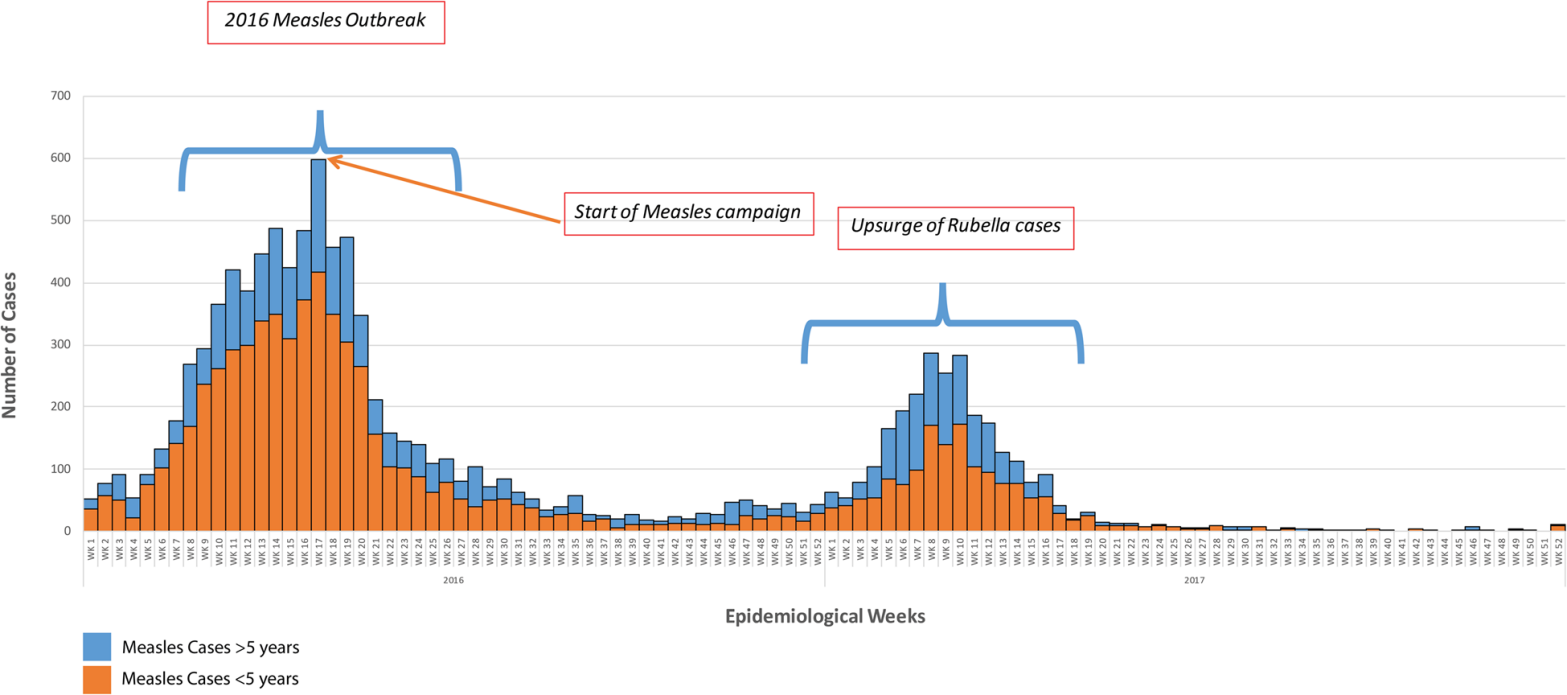
Epidemic Curve Showing Measles and Rubella Outbreaks in Sierra Leone 2016–2017

A suspected measles outbreak affecting 11/14 districts was promptly detected in the first quarter of 2016 when suspected cases increased tenfold over four months (Fig. 3). Descriptive analysis of data from suspected cases at the national level generated district and age specific attack rates which directed the response team to focus the vaccination campaign to children aged < five years beginning in the most affected districts. The measures taken were effective and led to a rapid decline in the number of reported cases. Laboratory confirmation, done on a small subset of the cases, helped confirm the causative agent during the outbreak. In February 2017, incorporating laboratory results of suspected measles cases helped differentiate an outbreak of rubella that may have otherwise been confused for measles based on the clinical presentation of the cases.

### Use of malaria surveillance to monitor trends in acute febrile illnesses

Malaria is endemic in all districts in Sierra Leone and is easily diagnosed using rapid diagnostic kits. In the revitalized surveillance system, each district submitted weekly summaries of the number of suspect malaria cases and the number of confirmed malaria cases. The MOHS used this data is used to generate trends in the malaria positivity rates for each district and to indirectly monitor the occurrence of febrile illness. Over time, the average malaria positivity rates and seasonality of malaria in Sierra Leone were established. Changes in malaria positivity were monitored as they were considered as a proxy for changes in occurrence of other febrile illness. Thus a reduction in malaria positivity beyond a given threshold in a given point in time, would indicate a possible increase in the occurrence of other acute febrile illnesses thus raising an alert that would then be investigated. Generating accurate malaria positivity trends was possible due to the high reporting rates in the revitalized IDSR system.

### Enhanced surveillance for Ebola virus disease

To improve case detection of suspected Ebola virus disease cases, the MOHS integrated enhanced Ebola virus disease surveillance into the routine IDSR. Thus, Ebola virus disease surveillance system was anchored within the IDSR system, utilizing the same healthcare workers, laboratory network, district surveillance officers and national officers as the IDSR system. Enhanced Ebola virus disease surveillance, was a form of syndromic surveillance system in which MOHS was alerted about cases of febrile illness that suspected to be due to Ebola virus disease. Samples are collected from suspected acute viral haemorrhagic fever cases, alive or dead and tested for Ebola virus disease. Enhanced Ebola virus disease surveillance, prompt reporting and control of suspected Ebola virus disease cases enabled the country to interrupt the transmission of Ebola virus since the outbreak was declared over in November 2015.

### Addressing maternal mortality through IDSR

During the Ebola virus disease outbreak in 2014–2015, the focus of the healthcare system shifted to control of the outbreak, at the expense of other service delivery areas, including reproductive health services. In the post Ebola recovery phase, the MOHS prioritized maternal and child health and thus sought to monitor the trends in maternal mortality by integrating reporting of maternal deaths through IDSR. In 2016, there were 218,818 live births reported through the health information system in Sierra Leone. Given the maternal mortality rate of 1165, the projected number of maternal deaths was 2549. The number of maternal deaths reported to the MOHS increased from 456 in 2015 to 706 in 2016, but was still 73% lower than the projected number. Almost all the reported deaths were audited and actionable recommendations provided. Whereas data incompleteness affected the validity of the mortality estimates, the system was useful in identification of weaknesses in antenatal and maternity care. This allowed the MOHS to identify health facilities with high burden of maternal deaths, the specific causes of death and prescribe specific interventions for reduction of maternal deaths in each health facility. The roll out of emergency obstetric care was a third element in ensuring an appropriate response to maternal deaths.

### The role of rapid response teams in outbreak investigation

Each district health team identified designated health care workers who were trained in rapid response. The teams consisted of clinicians, an epidemiologist/ district surveillance officer, laboratory technician, environmental health scientist and a health educator. Veterinary officers were incorporated during zoonotic outbreaks and industrial poisoning experts during suspected chemical event.

Reported outbreaks of notifiable diseases, unusual changes in surveillance data, reports of severe illness among healthcare workers, rumours of death or cases of illness in the community and unexplained death among animals were among the events that prompted an investigation by the rapid response team. Few exceptions included cases of viral haemorrhagic fever, unusual and emerging infections, or protracted outbreaks which required deployment of the national rapid response team to complement the response by the district rapid response team. In such instances, the emergency operations centre was activated to coordinate the response at the national level of the MOHS. In addition to investigation, national and district teams identified appropriate strategies to control the outbreaks, led the implementation of the strategies and wrote outbreak investigation reports.

### Preparedness to respond to outbreaks and other public health events

Response to outbreaks is more likely to be prompt and effective when the health system is prepared. The national preparedness plan for Sierra Leone outlined the roles and responsibilities for the Public Health Emergency Management Committees at the national and district levels. The membership of this committee was diverse, incorporating both technical and non-technical members including officials from relevant ministries.

The district public health emergency management committees developed and implemented district preparedness plans. They identified and planned for all possible emergencies in the district, mobilized resources, developed communication strategies and coordinated procurement of emergency material stockpiles. The emergency operations centre, located in the directorate of disease prevention and control, was tasked with the coordination of public health response activities during outbreaks and other public health emergencies. The emergency operations centre was also involved in continuous planning to ensure that the country was prepared to respond to emergencies.

### Monitoring implementation of IDSR

Close monitoring of IDSR activities was necessary as challenges in implementation of IDSR were expected. Supervisory visits in all districts began in February 2016, soon after the last district teams had been trained on IDSR. By July 2017, three countrywide visits were conducted. Improvement in IDSR reporting completeness and timeliness rates was partly attributed to the support supervision.

Two data quality assessments were conducted between August 2016 and July 2017. Overall, data collected through the surveillance system was of good quality and there was a modest improvement in accuracy from 95.3% in the first assessment to 97% in the second assessment. Over reporting was more common in large volume health facilities and most discrepancies were between the number of records in the health facility registers and the number of records entered in the monthly summaries. The findings of the data quality assessments were discussed in national surveillance quarterly meetings convened by the directorate of disease prevention and control and attended by all district medical officers and district surveillance officers. It was noted that the accuracy and completeness of data improved with time, most likely due to the periodic assessments and feedback.

## Discussion

### A functional IDSR system “from Ebola to Health”

As part of health systems recovery which was commonly referred to as the shift from “Ebola to Health”, a revitalized IDSR system with key performance indicator above the WHO AFRO targets was achieved. Recruitment and deployment of WHO international epidemiologists and public health experts to support MOHS at national and district level (2015–2017) helped in strengthening health systems through mentorship, knowledge & skill transfer. Starting 2016, these efforts were complimented by the US Centres for Disease Control and Prevention (CDC) supported Field Epidemiology Training Programme that equipped the frontline health workers with basic epidemiological skills. Partnership between WHO and CDC, department for international development, African development bank, multi partners trust fund and other partners made significant investments in IDSR during the recovery phase after the protracted Ebola virus disease outbreak. In the long term, there is a need for the MOHS to mobilize domestic financing for sustainability of the IDSR system.

Successful implementation of IDSR required proper planning, prioritized phased implementation, and political goodwill that provided additional impetus. Availability of the IDSR tools and guidelines, ICT infrastructure for data transmission, well trained workforce, and logistical support aided implementation. Lack of support functions for IDSR decreases the capacity of a country to perform IDSR core functions optimally [8].

The integration of enhanced Ebola virus disease syndromic surveillance into the IDSR increased efficiency while allowing for prompt detection and investigation of suspected Ebola virus disease cases. This contributed to interrupted transmission of Ebola Virus with the outbreak declared over in November 2015. Syndromic surveillance is an effective means of augmenting routine indicator based surveillance and meeting the International Health regulations on timely reporting of public health events of international concern [12]. Given that outbreaks of Ebola Virus disease occur frequently [13], strengthening public health surveillance should be prioritized especially in countries prone to Ebola virus disease outbreaks.

Inclusion of non-infectious conditions such as maternal death in the IDSR system, improved the utilization of surveillance personnel and systems thus increasing efficiency. The maternal mortality ratio in Sierra Leone is estimated to be 1165 maternal deaths/ 100,000 births and is among the highest in the world [14]. Overall deterioration of health services during the Ebola outbreak may have increased the maternal mortality ratio and thus there was a need to monitor the trends during the recovering period.

Measures of the usefulness of a surveillance system include the proportion of outbreaks that are detected through the surveillance system, the promptness of the response to suspected outbreaks and the duration taken to provide laboratory confirmation on the causative agent [7]. Based on the high proportion of outbreaks detected through the Sierra Leone IDSR system it appears to be highly sensitive. However, a reduction in the proportion of outbreaks detected through the IDSR system was observed over time and the reduction was attributed to a reduced number of WHO personnel in the districts. This fluctuation in performance indicates a potential threat in the sustainability of IDSR in Sierra Leone and calls for increased ownership by MOHS.

There was an apparent improvement in completeness and timeliness of IDSR reporting after shifting to electronic data transmission, that is likely due to ease of data collection and transmission at the district level. This is contrary to a study of performance of IDSR in Kenya, where 41% of sampled health facilities had not submitted weekly IDSR reports in the preceding 12 weeks and where health care workers preferred electronic transmission of reports. The shift to e-IDSR was hampered by poor internet connectivity, especially in the remote districts. The improvement in IDSR indicators attributed to the shift to e-IDSR can justify the use of resources to extend electronic data collection and transmission up-to the health facility level. Data quality assessments revealed over reporting of cases in the IDSR system although data accuracy improved with time. The most likely contributor to poor data quality was the paper based reporting that required transcription of data from various sources. This is corroborated by the improvement in data quality with the shift to e-IDSR. Weekly epidemiological bulletins and regular national surveillance meetings are mechanisms that can be used to provide feedback to all relevant stakeholders and ultimately improve quality of surveillance data. Lack of feedback from the higher reporting levels and lack of supportive supervision have been identified as gaps in the implementation of IDSR in India and Ghana [15, 16]. Regular supportive supervision and data quality assessments may have improved IDSR data accuracy and completeness over time.

## Conclusions

The MOHS, Sierra Leone, supported by various partners successfully revitalized IDSR, as part of a wider health sector recovery plan. Capacity building of health care workers, availability of materials, and infrastructure to support implementation, frequent supervision and feedback contributed to the successful implementation of IDSR. Subsequently, an early warning system was established that facilitated interrupted transmission of the Ebola virus disease outbreak in November 2015. The usefulness of IDSR in outbreak detection and control builds the case for global investment in IDSR activities as it ultimately leads to increased global health security. In Sierra Leone, MOHS needs to focus on improving laboratory diagnosis, increasing domestic financing of surveillance activities and institutionalization of data quality assessments at the district level.

## Additional file

Additional file 1: Weekly Epidemiologic Bulletin, Ministry of Health and Sanitation, Sierra Leone, 2017. A weekly summary of cases of priority diseases reported through the public health surveillance system in Sierra Leone during epidemiologic week 52, 2017. The summary also shows the intra-district reporting rates and timeliness in reporting for all the districts in Sierra Leone. (PDF 892 kb)

## Abbreviations

CDC: United States Centers for Disease Prevention and Control
e-IDSR: Electronic Integrated Disease Surveillance and Response system
IDSR: Integrated Disease Surveillance and Response
WHO: World Health Organization
