# Supplementary material for: Revitalization of integrated disease surveillance and response in Sierra Leone post Ebola virus disease outbreak
Source: BMC Public Health. 2019 Apr 2;19:364. doi: 10.1186/s12889-019-6636-1 (PMC6444503; doi:10.1186/s12889-019-6636-1)
Supplement: Supplementary file 1 — Weekly Epidemiologic Bulletin, Ministry of Health and Sanitation, Sierra Leone, 2017. A weekly summary of cases of priority diseases reported through the public health surveillance system in Sierra Leone during epidemiologic week 52, 2017. The summary also shows the intra-district reporting rates and timeliness in reporting for all the districts in Sierra Leone. (PDF 892 kb) [file 12889_2019_6636_MOESM1_ESM.pdf]

MINISTRY OF HEALTH AND SANITATION

Republic of Sierra Leone

World Health Organization

INTEGRATED DISEASE SURVEILLANCE AND RESPONSE (IDSR)

Volume 10, Issue 52 **WEEKLY EPIDEMIOLOGICAL BULLETIN** **Week 52: 25 December – 31 December, 2017**

Highlights of the week

- All the 14 districts submitted timely reports to the national level this week using the eIDSR platform. The average district reporting timeliness rate is 99% for the period of week 1 to 52.
- Countrywide, 94% of health facilities (HFs) submitted IDSR reports to the district level this week.
- In the week, only 36% (3080/8,595) of the trained Community Health Workers (CHWs) in 4 out of the 9 districts currently implementing Community Based Surveillance (CBS) submitted reports to the health facility level.
- One case of Acute Flaccid Paralysis (AFP) reported from Kono district. The DRRT investigated the case , stool samples collected and sent to CPHRL for confirmatory testing.
- Nine maternal deaths were reported in the week, a decrease from 5 deaths reported in the previous week. The deaths were from Bo (1), Port Loko (2), Tonkolili (2) and Western Area Urban (4) districts.
- Eleven Measles cases were reported in the week. The cases were from PortLoko (8), Western urban (2) and Koinadugu (1) districts. The DRRT from the respective districts investigated the cases, blood samples collected and sent to CPHRL for confirmatory testing.
- A total of 14 animal bites cases were reported in the week, a decrease from 15 cases reported in week 51. Eleven out of the 14 districts reported cases this week with Bo (3) and Western Area Rural (2) districts reporting the highest, the rest of 11 districts reported 1 animal bite each.
- Clinical Malaria cases totaling to 41,249 were reported in the week. Of these, 41,136 (99.7%) were tested, out of which 22,399 were confirmed positive, amounting to a positivity rate of 54.5%. A total of 75 deaths from confirmed Malaria cases were reported in the week.
- Reported Dysentery (bloody diarrhea) cases reduced to 23 from 25 in the previous week. Majority of the cases were from Bonthe (5) Kenema (4) Pujehun (4), and PortLoko (3) districts.
- A total of 389 cases of Severe malnutrition were reported during the week, a reduction from 424 in the previous week. The highest number of cases were reported from Port Loko (65), Pujehun (45), Moyamba (35), Kailahun (33), Bo (32) and Western Area Urban (32) districts.

Intra-District Health Facility Reporting Rate for Week 52	
Bombali (100%)	Kono (97%)
Bonthe (100%)	Western Area Urban (97%)
Kambia (100%)	PortLoko (96%)
Tonkolili (100%)	Bo (95%)
Western Area Rural (100%)	Koinadugu(91%)
Kailahun (100%)	Kenema (79%)
Moyamba (97%)	Pujehun (72%)

Suspected Typhoid fever Surveillance Update

A total of 81,598 Typhoid Fever cases with 53 deaths have been reported through the weekly IDSR surveillance system in weeks 1 to 52 of 2017, compared to 75,097 suspected cases with 317 deaths reported in same period in 2016. Majority of the cases this year have been reported from Western Area Urban (22,177), Kono (15,354) and Bombali (10, 112). For the rest of the districts cases ranged from 929 in Moyamba to 5,799 in Port Loko

There is apparent over-diagnosing of suspected Typhoid Fever cases and all DHMTs are argued to keep sensitizing health workers on the proper use of Typhoid Fever case definition as per IDSR Technical Guidelines especially during the monthly In-Charges meetings.

Figure 1: Average Intra-District Health Facility Reporting Rate For Week 1 to 52, 2017

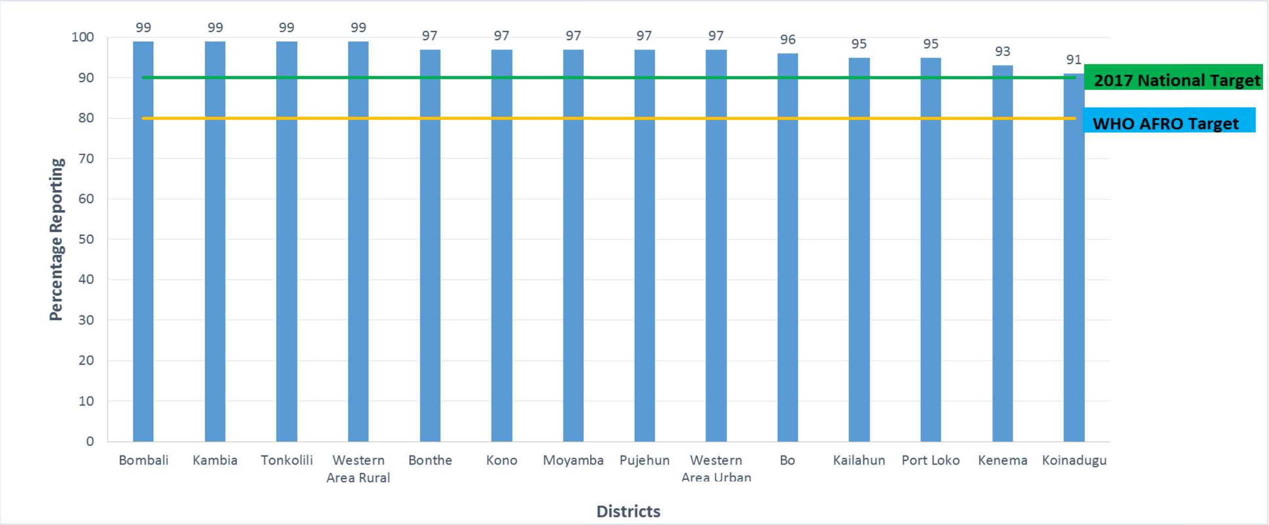

In the period of week 1 to week 52 of 2017, all districts achieved the national intra-district health facility reporting rate target of ≥90%*. On average, 97% of the expected health facility reports have been submitted to the district level since the beginning of the year

**The country revised the weekly IDSR reporting rate (completeness) target from ≥80% to ≥90% since week 7 of 2017*
